# Midwifery

**Published:** 1837-07

**Authors:** 


### MIDWIFERY.

1. *Observations on the Artificial Dilatation of the Mouth of the Womb during Labour, and upon instrumental Delivery, &c.* By Robert Collins, m.d. &c. *Dublin.*
2. *An Inquiry into the Management of the first Stage of Labour.* By E. W. Murphy, m.d., *Dublin.*

Both these articles, written by accoucheurs of talent and experience, are published with the view of controverting and counteracting the doctrine so strongly advocated in the recent publications of Dr. Hamilton, of the propriety of inducing labour artificially at a certain period after the natural commencement of the process. As we have noticed them in a review of the Second Part of Dr. Hamilton's

"Observations," (prepared for the present Number, but postponed for want of room,) we advert to them in this place, merely to express our opinion of their great value and of the soundness of the doctrine advocated in them; and to assure our readers that they will find them worthy of careful perusal.

*Dublin Journal. March and May,* 1837.

---

*On the length of the Umbilical Cord, and its mechanical Influence upon Parturition.* By F. CHURCHILL, M.D., *Dublin.*

THIS short paper is valuable, both in a physiological and practical point of view. After stating the opinions of various writers on the length of the cord, and the supposed effect of this in modifying labour, Dr. C. gives a statistical view of all the exact admeasurements of the funis which he has met with, including 212 cases of his own. The total number of cases is 391. The shortest cord in these was twelve inches, and occurred in six cases; the longest was fifty-four inches, and only occurred in one case; the length which occurred most frequently was eighteen inches, and the next in frequency twenty-four inches. Out of 190 cases, Dr. C. found the cord round the neck in 52; the shortest cord found so disposed was eighteen inches, and occurred twice in 75 cases. The cord was never under two feet when coiled twice round the neck; nor under three when coiled thrice round. It was coiled four times round in one case where it was three feet, and also in one case where it was fifty-four inches. Whenever it exceeded two feet, it was generally round the neck.

From these facts Dr. C. concludes, that there is less risk from the twisting of the cord round the neck than is commonly believed. As labour may be most safely conducted when the cord is thirteen inches long, and as even ten inches may suffice when the placenta is extended laterally; consequently, since, in all the examples where the cord was round the neck, there were, at least, thirteen inches free, no risk need be apprehended, in ordinary circumstances, from such a state of things.

We give the practical application of the whole of the observations in Dr. Churchill's own words.

"By almost all authors we are impressed with the necessity of untwisting the coil around the neck, by slipping it over the head or shoulders, in order to give the child the benefit of the full length of the cord. In many cases this is very difficult; in some, it is impossible. We have seen that in by far the majority of instances this is perfectly unnecessary, as no evil consequences can follow, there remaining, allowing for the coil, an adequate portion of the cord free. The cord should in all cases be drawn down a little, to relieve the stress upon it, and to loosen the part round the neck; but, except in a very few cases, more will not be necessary."

*Dublin Journal. March* 11, 1837.

